# The influence of a dicationic surfactant on the aggregation process of the IVAGVN peptide derived from the human cystatin C sequence (56–61)†

**DOI:** 10.1039/d4ra08377f

**Published:** 2025-01-31

**Authors:** Julia Ludwiczak, Emilia Iłowska, Michalina Wilkowska, Aneta Szymańska, Marek Kempka, Maria Dobies, Kosma Szutkowski, Maciej Kozak

**Affiliations:** a Department of Biomedical Physics, Adam Mickiewicz University Poznan Poland; b Department of Organic Chemistry, University of Gdansk Gdansk Poland emilia.ilowska@ug.edu.pl; c Department of Biomedical Chemistry, University of Gdansk Gdansk Poland; d NanoBiomedical Centre, Adam Mickiewicz University Poznan Poland

## Abstract

Human cystatin C (hCC) undergoes domain swapping and forms amyloid structures. Steric zipper motifs, which are important for hCC fibrillization, have been identified and studied in our previous work. In the present study, we analysed the influence of the selected dicationic surfactant (a derivative of dodecylimidazolium chloride: 3,3′-[α,ω-(dioxahexane)]bis(1-dodecylimidazolium)dichloride) on the structure of the aggregates formed by one such fragment, a peptide with the sequence IVAGVN, corresponding to residues 56–61 in the full-length protein. Changes in the secondary structure of the peptide induced by the surfactant were studied using circular dichroism (CD) and FTIR, and the aggregates were characterised using microscopic techniques (AFM and TEM) and NMR.

## Abstract 2

Research of the effect of the dicationic gemini surfactant (3,3′-[1,8-(2,7-dioxahexane)]bis(1-dodecylimidazolium) dichloride (C6IMIC12) on the fibrillation process of the IVAGVN peptide using microscopic, spectroscopic and molecular docking techniques.

## Graphical abstract

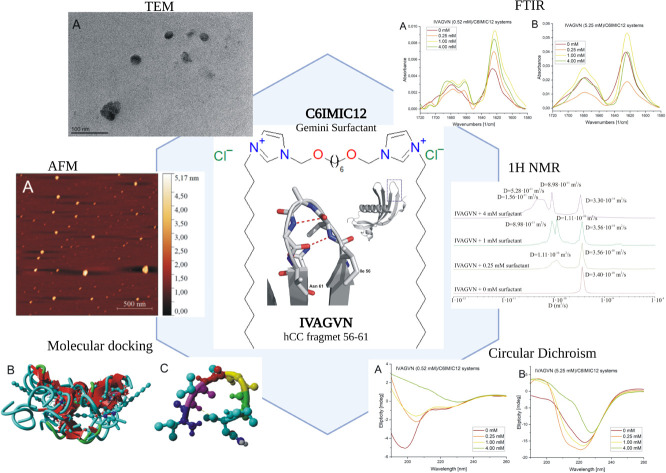

## Introduction

**Fig. 1:**
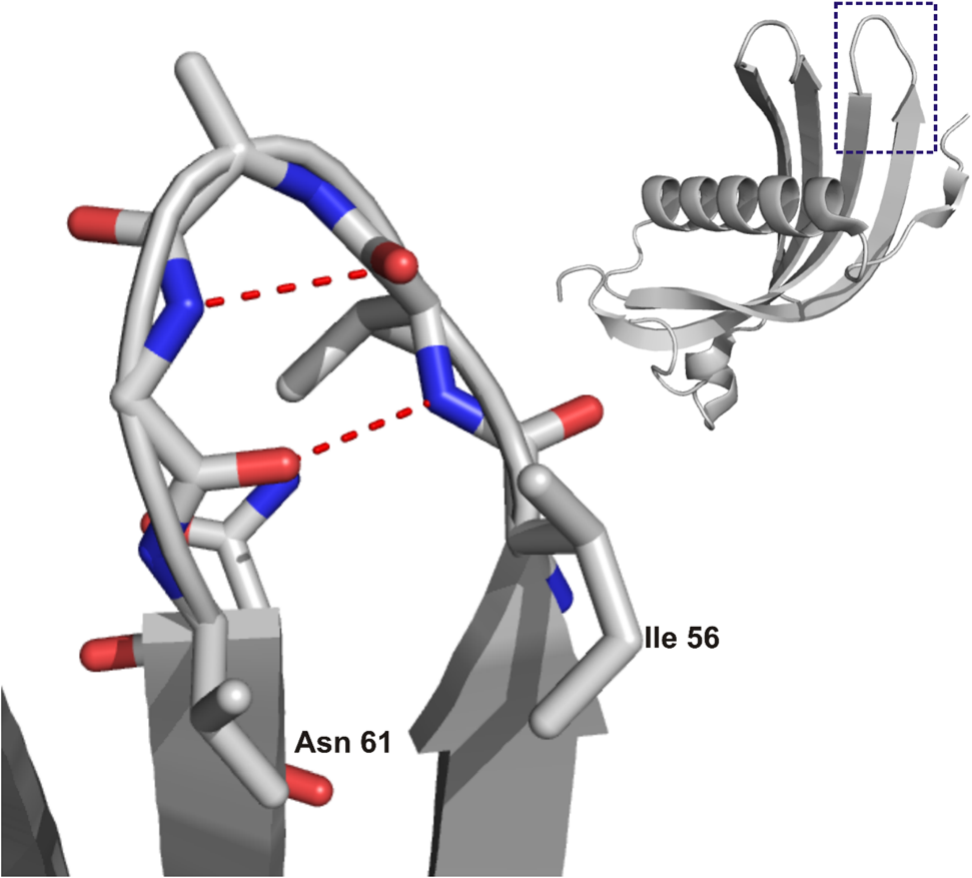
Structure of HCC (right) with a marked region of steric zipper motif and zoom (left) on the IVAGVN sequence. The image was prepared based on the crystal structure of the HCC monomer (PDB ID: 3GAX^20^) using NGL.^21^

Long-term development of numerous neurodegenerative diseases is associated with the gradual misfolding of disease-related proteins or peptides, followed by their aggregation and accumulation in the brain.^1,2^ The major degradation system of misfolded proteins is the ubiquitin-proteasome system (UPS). However, in pathological states, misfolded proteins exhibit resistance to proteolysis, resulting in their gradual accumulation as amyloid deposits in tissues and organs.^3^ In addition to proteins and peptides commonly related to neurodegenerative disorders, such as Alzheimer's (Aβ peptide) or Parkinson's disease (synuclein), there is a lesser-known protein that forms amyloid deposits in the human brain, namely, cystatin C (hCC).^4–6^ Under physiological conditions, this protein is responsible for the inhibition of proteolytic processes catalysed by cysteine proteases.^7–10^ A natural mutation in the human cystatin C sequence at position 68, where leucine residue is replaced by glutamine, leads to massive deposition of amyloid aggregates in brain vessels.^4,11^ Studies of the hCC structure, including wild type protein and several point mutants, allowed us to propose that the formation of higher oligomers and aggregates most likely occurs through the mechanism of domain swapping,^12–15^ which is associated with specific structural fragments^16–18^ of this protein. The most significant one is flexible loop L1, encompassing residues 56–61. Notably, this region is also part of a longer sequence that Tsiolaki and co-workers^19^ have proposed as a fragment of hCC involved in its aggregation. The peptide CysC2 (sequence: VAGVNYFLD, residues 56–65), which, in the full-length protein, forms loop L1 (plus surrounding fragments of β-strands; Fig. 1 (ref. 20 and 21)), can form fibrillar aggregates. Subsequent studies conducted by Iłowska and co-workers resulted in the “splitting” of this sequence into shorter fragments with diverse tendencies to form fibrils or aggregates.^22^ One of these peptides, IVAGVN (residues 56–61), was used in the present study. The kinetics of amyloidogenic processes is closely linked to the influence of several physicochemical parameters, such as pressure, temperature, and pH value.^23,24^ Furthermore, protein molecules in cells and in extracellular spaces are not isolated. They are located in a crowded environment where they interact with many different types of molecules, such as lipids and surfactants.^25^

Gemini surfactants from the group of oligomeric surfactants are used in nucleic acid transfer systems owing to their effective complexation properties coupled with relatively low cytotoxicity.^26–30^ Recently, they have gained considerable attention owing to their unique structural characteristics and potential uses in biomedicine. These surfactants have two hydrophilic head groups and two hydrophobic tails linked by a spacer, which distinguishes them from conventional surfactants. They exhibit lower critical micelle concentrations (CMC) and enhanced surface activity.^31^ These properties render them particularly suitable for applications involving protein interactions, including drug delivery systems and the stabilization of biomolecules.^32^ For instance, research by Al-Dulaymi *et al.* emphasised the design of peptide-modified gemini surfactants that exhibit high transfection efficiency and low cytotoxicity, indicating their potential for effectively delivering peptide sequences.^33^ Recent studies have highlighted the ability of peptide-modified gemini surfactants to interact with aggregation-prone regions of proteins, such as the amyloid-β (Aβ) peptide, which is relevant in the context of neurodegenerative diseases. Molecular docking studies conducted by Narsineni *et al.* demonstrated that gemini surfactants can effectively bind to specific peptide regions, suggesting their potential role in modulating protein aggregation processes.^34^ This is of particular interest for peptides derived from the 56 to 65 residue region of hCC because these sequences may play crucial roles in the protein's functional and structural integrity, as well as its stability and aggregation behavior.^35^ Moreover, the structural characteristics of gemini surfactants allow for enhanced interactions with small peptides derived from proteins. The interactions between gemini surfactants and small peptides can be influenced by the spacer length and hydrophobic tail structure. Sonu *et al.* explored the effects of different spacer groups and hydrocarbon tail lengths on the formation of mixed micelles, revealing that these structural variations significantly impact the hydrophobicity and aggregation behavior of surfactants.^36^ This finding suggests that the design of gemini surfactants can be tailored to optimize their interactions with specific peptide sequences, including those derived from hCC.

Furthermore, depending on the concentration, the surfactant can increase or inhibit the protein aggregation process. The influence of the dicationic gemini surfactant hexamethylene-1,6-bis-dodecyldimethylammonium dibromide and monomeric cationic surfactant dodecyltrimethylammonium bromide on the aggregation behaviour of the amyloid β-peptide (Aβ1–40) studied by Li *et al.*^37^ showed that at low concentrations, the surfactant supports aggregation, while at higher concentrations, it leads to destabilization of fibrillar structures, with eventual micellar and globular forms observed above CMC and critical aggregation concentrations (CACs) of the surfactant. The unfolding and refolding processes of model proteins in the presence of gemini surfactants and cyclodextrins were also studied by Kumari *et al.*^38^ These studies show that at low concentrations, surfactants facilitate fibrillar aggregation, while at higher concentrations, they disrupt the structure of fibrillar aggregates or unfold proteins. In the present research, we focused on the interaction of the model IVAGVN peptide with the dicationic gemini surfactant (3,3′-[1,8-(2,7-dioxahexane)]bis(1-dodecylimidazolium)) dichloride (C6IMIC12). The choice of the surfactant was not accidental because its chemical structure overall mimics a biological lipid environment. The peptide, on the other hand, was chosen because it has good solubility while at the same time demonstrating the ability to fibrillate, making it easier to control the processes involved.

The objective of this study is to investigate the impact of gemini surfactants on the structural behaviour of the aggregation-prone fragment of cystatin C-IVAGVN peptide, with a particular focus on the influence of surfactant concentration on the peptide's secondary structure and aggregation tendencies. We seek to gain insight into how surfactants influence the initial stages of structural transitions that may precede or prevent fibrillization. Such insights are vital for understanding how surfactants can either stabilize or destabilize peptide assemblies, with the potential to provide new strategies for controlling amyloid fibril formation. These findings may facilitate the development of therapeutic agents that inhibit or modulate pathological fibrillization processes, thereby contributing to efforts in combating neurodegenerative diseases.

## Results

### Molecular docking

**Fig. 2:**
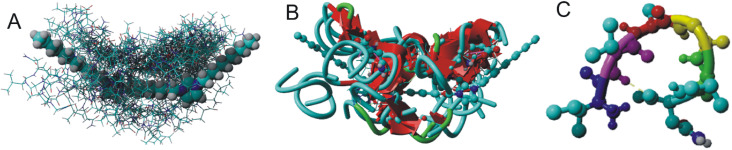
A molecule of gemini C6IMIC12 surfactant (greyed out) with nonspecific binding sites of IVAGVN: (A) ball-stick representation; (B) receptor–ligand representation with β-sheets depicted in red; and (C) peptide structure as derived from omega-fold. The hydrogen bond in the β-turn is between ^57^Val and ^61^Asn.

Fig. 2 depicts the molecular structure of the C6IMIC12 gemini surfactant and the numerous non-specific binding sites for the peptide. Each docking site represents regions where the peptide can form transient interactions with the surfactant primarily through hydrophobic contacts with the alkyl chains or electrostatic interactions with the charged head groups. These interactions are dynamic and may vary depending on the peptide's concentration and the surfactant's critical micelle concentration. Although the binding energies vary from Δ*G* −9 to −5 kJ mol^−1^ indicating either low or moderate binding, the interaction is sufficient to influence the peptide's conformation and promote changes in secondary structure or aggregation states (Fig. 2B). The β-sheet content for all peptide molecules in Fig. 2B varies from 45% to 63% (the remainder consists of turns and coils). The naturally occurring state of the peptide does not exhibit β-sheets (Fig. 2C). This behaviour is particularly relevant for understanding how surfactants modulate peptide states in crowded or biologically mimicking environments, providing insights into the early stages of structural transitions that may precede fibrillization.

### Molecular dynamics simulations and the impact of peptide aggregation on the secondary structure

**Fig. 3:**
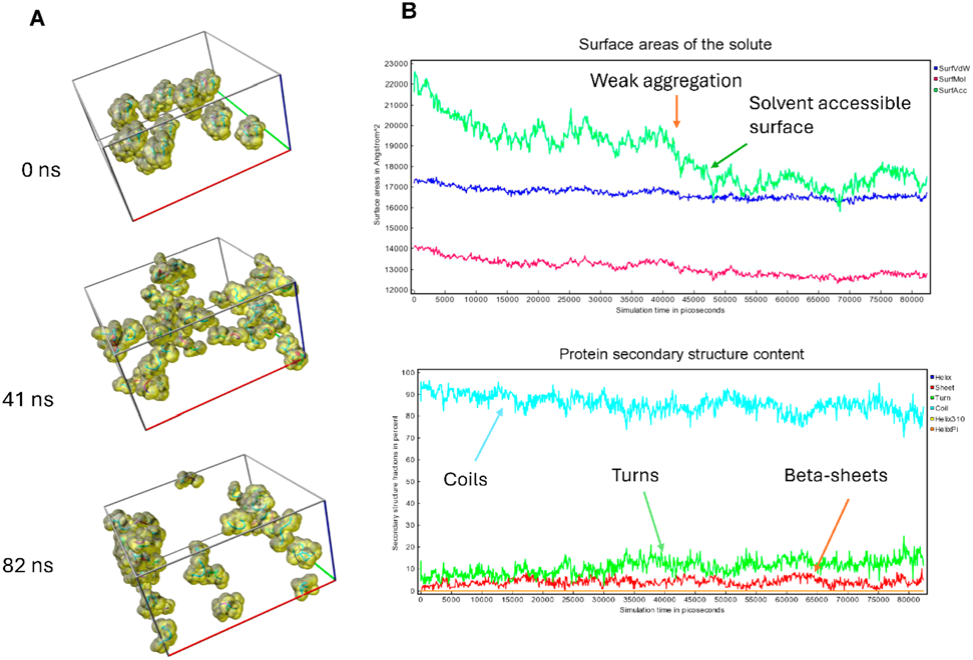
Molecular dynamics simulation was conducted for 45 molecules of IVAGVN peptides, and the following results were obtained. (A) Snapshots from the simulation with a ball-stick representation and visualization of a solvent-accessible surface. (B) Top figure: MD analysis for the 0–80 ns interval showed the changes in the molecular surface (lowest), van der Waals surface (middle), and solvent-accessible surface (highest values).

To investigate the potential impact of aggregation or molecular crowding, as well as the effect of an anisotropic molecular structure on the possible anisotropy of diffusion coefficients, molecular dynamics simulations in water were performed. Fig. 3A shows simulation snapshots of 45 peptide molecules. Fig. 3B illustrates the analysis of the changing surface area throughout the simulation (molecular surface, VdW surface and solvent-accessible surface), as well as changes in secondary structures, such as coils, turns, and β-sheets content. Coil-type structures strongly predominate, but it can also be observed that β-sheets start to form albeit in a random manner. It is noteworthy that as the solvent-accessible surface decreases, peptides begin to loosely aggregate, forming unstable aggregates that most likely influence the secondary structure. Specifically, there is a weak decrease in coil-type structures, accompanied by a slight increase in turns and β-sheets. The shape of peptide molecules is anisotropic, which affects their mode of aggregation and the system partially orders itself, potentially contributing to the formation of amyloid aggregates. As illustrated in Fig. S1,† the overall dependence of the MSD on time is shown, along with linear fits to the diffusion coefficients according to the Einstein–Smoluchowski equation, MSD = 〈*z*^2^〉 = 6*Dt*, where mean-square displacement (MSD) is a function of diffusion coefficient *D* and time *t*. To analyse the potential effect of molecular anisotropy on the diffusion coefficient, the MSD was decomposed into individual components (MSD_*xx*_, MSD_*yy*_, MSD_*zz*_, MSD_*xy*_, MSD_*xz*_, and MSD_*yz*_). After the diagonalization, the major diffusion components of peptide in water (*λ*) were determined, *e.g.* anisotropy of displacement in the molecular frame of reference. Then, the average diffusion-coefficient values were derived by averaging the eigenvalues. The result is similar to the linear fit shown in Fig. S1.† In conclusion, the simulations indicate that significant interactions between molecules start to appear at around 40 ns, influencing both the values of the diffusion coefficients and the peptide secondary structures.

### Impact of peptide concentration on the secondary structure and microstructure of peptide

#### Microscopy research – transmission electron microscopy and atomic force microscopy

**Fig. 4:**
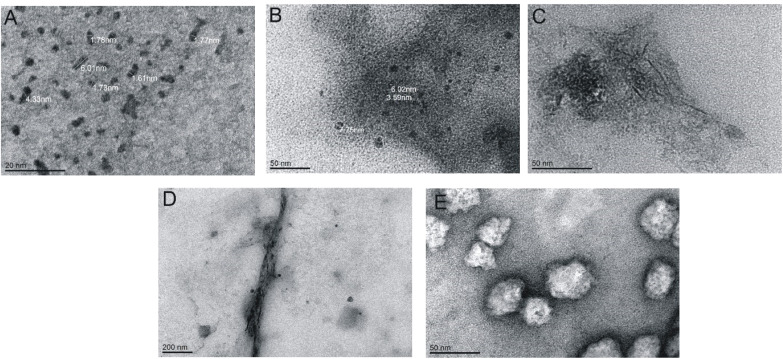
TEM images for various concentrations of peptide: (A) 0.52 mM, (B) 1.75 mM, (C) 2.00 mM, (D) 3.50 mM and (E) 5.25 mM.

Many physicochemical factors can influence the aggregation process of a peptide or protein.^39–42^ To describe the aggregation properties of the IVAGVN peptide, we first assessed whether the morphology of aggregates depends on peptide concentration using transmission electron microscopy (TEM) and atomic force microscopy (AFM). The TEM images obtained at different peptide concentrations are presented in Fig. 4. At low peptide concentrations, small, mostly circular aggregates with diameters of around 2 nm were formed (Fig. 4A and B). They seemed to show a tendency towards self-association, resulting in the formation of slightly elongated structures (Fig. 4C). Increasing peptide concentrations enhanced this clustering propensity, leading to large aggregates with diameters of around 50 nm. Among them, short fibrils can be observed with a length of *ca.* 23 nm for a concentration of 2.60 mM and significantly longer fibrils of about 1 μm at a concentration of 3.50 mM (Fig. 4C and D). At the highest concentration, the formation of spherical forms was repeatedly observed, which cannot be unambiguously interpreted, given the presence of fibrils at lower concentrations of the peptide (Fig. 4E).

**Fig. 5:**
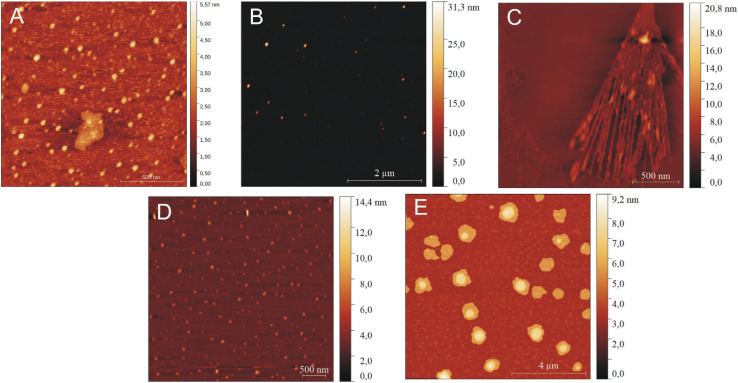
AFM images for various concentrations of peptide: (A) 0.52 mM, (B) 1.05 mM, (C) 2.60 mM, (D) 3.50 mM, and (E) 5.25 mM.

To characterize independently the morphology of the formed peptide structures, AFM experiments were performed. At low peptide concentrations, IVAGVN forms spherical structures characterized by a diameter of a few nanometres (Fig. 5A), which further increases for higher peptide concentrations (Fig. 5B). As the concentration increased, the globular structures transitioned into increasingly thicker fibrils (Fig. 5C).

A further increase in concentration leads to the formation of larger irregular aggregates with a diameter of around 50 nm. At the highest IVAGVN concentration, the aggregates begin to overlap, forming large spherical forms (Fig. 5E), with a diameter of around 1 μm.

#### Nuclear magnetic resonance ^1^H NMR

**Fig. 6:**
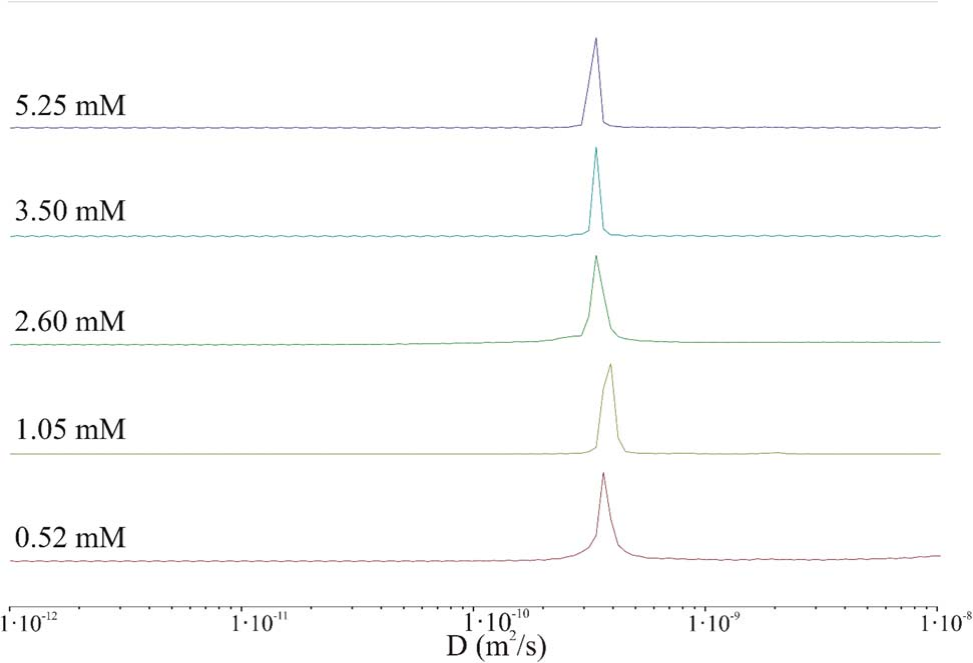
Protection of distribution of diffusion coefficient derived from 2D DOSY spectra for various peptide concentrations: 0.52 mM (red), 1.05 mM (light green), 2.60 mM (green), 3.50 mM (blue), and 5.25 mM (purple).

DOSY NMR measurements were performed to determine the structural and dynamic parameters (hydrodynamic radius-*R*_h_, diffusion coefficient-*D*) of the IVAGVN peptide in deuterated PBS at various concentrations. The obtained spectra are shown in Fig. 6 and S2 in the ESI Material.† The values calculated for the diffusion coefficient and hydrodynamic radius are presented in Table 1.

**Table 1:** Diffusion coefficients and hydrodynamic radius for various concentrations of the IVAGVN peptide

IVAGVN concentration [mM]	Diffusion coefficient *D* × 10^−10^ [m^2^ s^−1^]	Hydrodynamic radius *R*_h_ [nm]	Ellipsoid semi axis [nm]		Dimensions of aggregates in TEM [nm]
			*a*-Semi-major axis	*b*-Semi-minor axis	
0.52	3.40	0.64	2.1	0.16	1.78–2.00
1.05	3.50	0.61	2.1	0.14	2.00–6.50
2.60	3.46	0.63	2.1	0.15	2.00–6.50
					48.00–50.00^a^
3.50	3.46	0.63	2.1	0.15	2.60
5.25	3.46	0.63	2.1	0.15	45.00–49.00^a^

a Size of the whole aggregate or micelles.

The results clearly indicate that irrespective of the peptide concentration, both the diffusion coefficient and the hydrodynamic radius calculated using the Stokes–Einstein relation remain almost constant. Based on this, it can be concluded that the DOSY experiment performed under the selected conditions (incubation time, temperature, concentration, pH) does not confirm the presence of aggregated peptides in the sample. In fact, very large aggregates (hundreds of nm or larger) may be present in the sample but could be virtually undetectable by liquid NMR.^43^ The shape of the IVAGVN peptide can be approximated as an ellipsoid of revolution with semi-axes *a* and *b* = *c*. Assuming a tumbling motion of the peptide and fixing the length of the long axis at 2.1 nm, which is equivalent to the geometrical length of a 6-membered residue, the calculated^44^ lengths of other axes are presented in Table 1. Both approximations were performed by keeping the value of molecule volume *V*_IVAGVN_ = 0.23 nm^3^, as calculated from geometric assumptions.^45^

Notably, if we calculate the hydrodynamic radius directly from the molecular structure, we obtain a much smaller theoretical value of the hydrodynamic radius of around 0.17 nm. This value is obtained given the solvent accessible volume of around 1.45 nm^3^ and the solvent accessible surface area of 8.45 nm^2^. The hydrodynamic radius calculated based on NMR diffusion coefficients shown in Table 1 is rather large *ca.* 0.60 nm. Accordingly, the observed diffusivities correspond rather to small aggregates that appear irrespective of concentrations (MD simulations and MSD analysis in Fig. S2†). The diffusion coefficient in such a regime of weak interaction, obtained from simulations in water for a time of 82 ns, is about 2.50 × 10^−10^ m^2^ s^−1^. Furthermore, the diffusion is anisotropic and can be characterized as a diffusion tensor (Fig. S2, ESI Material†). This behaviour has a pronounced value for the aggregation and induction of amyloid growth. The diffusion coefficient of the order of 3.00 × 10^−10^ m^2^ s^−1^ may correspond to small aggregates that interact with each other but do not form large clusters/aggregates (Fig. S2, in ESI Material†). The diffusion coefficient in such a regime of weak interaction, obtained from simulations in water for a time of 82 ns, is about 2.50 × 10^−10^ m^2^ s^−1^. Furthermore, the diffusion is anisotropic and can be characterized as a diffusion tensor (Fig. S2, ESI Material†). This behaviour has a pronounced value for the aggregation and induction of amyloid growth. The appearance of larger aggregates may be detected at much larger time scales. When large aggregates are formed, the *D* parameter starts to decrease to 1.20 × 10^−10^ m^2^ s^−1^ but still cannot be associated with stable aggregates. These dimensions can be associated only with the diameters of the smallest spherical forms present in TEM and AFM images recorded at low peptide concentrations. Starting from a concentration of 2.60 mM, where fibrils and larger aggregates are observed in microscopic images, the diameters of the particles are definitely larger than suggested by calculations from NMR studies. This may suggest that the aggregates recorded microscopically are formed by the clustering/aggregation of smaller spherical forms, and similar to Hjalte *et al.*, we were unable to detect them.^43^ Another explanation could be the differences in the experimental set-up. For TEM and AFM, the peptide was dissolved in a phosphate buffer, while the NMR spectra were recorded for the aqueous peptide solution, in which the aggregation and fibrillization processes occurred slower.

#### Circular dichroism

**Fig. 7:**
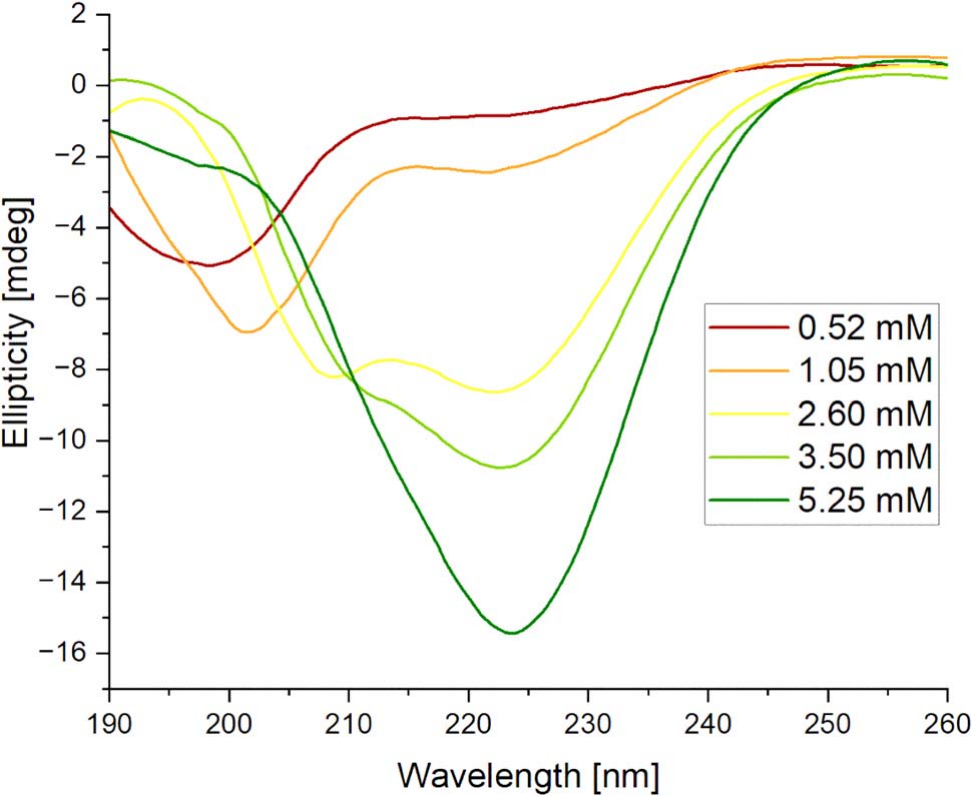
Circular dichroism (CD) spectra for IVAGVN recorded at various concentrations.

The effect of peptide concentration on its conformation was studied by circular dichroism (CD). The obtained results are presented in Fig. 7. As the peptide concentration increased, two significant changes in the secondary structures were observed. First, there was a transition from a random coil (RC) to an α-helical conformation at concentrations of 2.60 mM (from 7.8% to 16.6%; see Table S1, ESI Material†) and 3.50 mM (14.9%), followed by a shift to a β-sheet at the highest concentration. In the α-helical state, the CD spectrum displayed a maximum of around 190 nm and two characteristic minima at 205 nm and 220 nm. In contrast, the β-sheet exhibited a deep minimum at 220 nm but lacked or showed minimal signal in the 190 nm region.^46^ A similar phenomenon was previously observed with α-synuclein, where conformational changes were driven not by concentration but by incubation time. The researchers proposed that the appearance of the helical structure indicates a transient intermediate state, which also seems to occur with our peptide. This transient state may act as a structural precursor, facilitating the eventual formation of stable β-sheet-rich aggregates.^47^

### Impact of peptide and surfactant concentrations on the secondary structure and microstructure of peptide aggregates

#### Microscopic research – transmission electron microscopy and atomic force microscopy

**Fig. 8:**
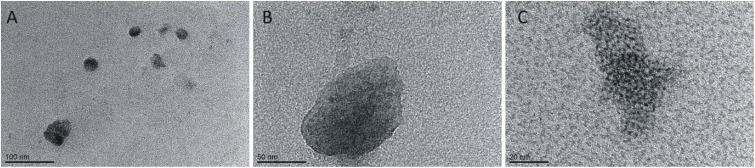
TEM images for various concentrations of C6IMIC12 surfactant: (A) 0.25 mM, (B) 1.00 mM, (C) 4.00 mM and constant peptide concentration (0.52 mM).

**Fig. 9:**
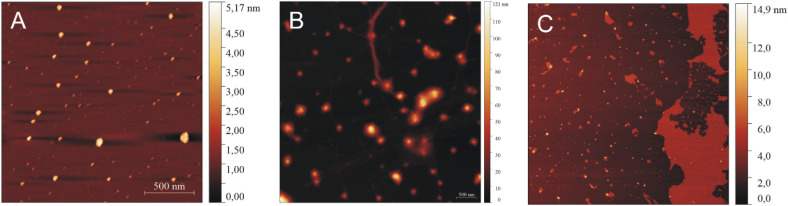
AFM images of IVAGVN (0.52 mM) with various C6IMIC12 surfactant concentrations: (A) 0.25 mM, (B) 1.00 mM and (C) 4.00 mM.

The aggregation of peptides and proteins can be significantly influenced not only by their concentration, the ionic strength or pH of the environment but also by the interactions with other compounds in the solution.^41^ Amphiphilic molecules can modulate the aggregation behaviour of some proteins and even induce a disaggregation process.^48^ Therefore, in the second part of our study, we analysed the influence of a cationic gemini surfactant on the aggregation of the IVAGVN peptide. However, considering the previously described varying behaviour of the IVAGVN peptide at different concentrations, particularly its ability to form morphologically diverse structures, we decided to analyse the behaviour of the peptide at two opposite concentrations (0.52 mM and 5.25 mM) in the presence of a surfactant at concentrations of 0.25 mM, 1.00 mM and 4.00 mM. Relating this to molar ratios, in the case of a lower peptide concentration (0.52 mM), the distributions were 2 : 1, 1 : 2 and 1 : 8. For high peptide concentrations, the corresponding distribution was 20 : 1; 5 : 1 and 3 : 1, respectively. In the TEM (Fig. 8) and AFM (Fig. 9) images recorded for peptides at low concentrations in the presence of varying surfactants, different aggregates were observed. In the case of a system at the lowest surfactant concentration, circular aggregates with a 20–30 nm diameter were present in the sample (Fig. 8A). These aggregates exhibit some tendency to self-associate to form larger, irregular forms (Fig. 8A). The increase in surfactant concentration appears to enhance this process (Fig. 8B). At the highest C6IMIC12 to peptide ratio, these structures seemed to be partially dissolved/disaggregated (Fig. 8C).

In AFM imaging of samples with a peptide to surfactant molar ratio of 2 : 1, circular aggregates with a diameter of approximately 30 nm were observed (Fig. 9A). The size of aggregates increased for a molar ratio of 1 : 2, where circular aggregates with diameters ranging from 40 to 50 nm were formed alongside thin fibrils (Fig. 9B). At the highest C6IMIC12 concentration, large IVAGVN aggregates and fibrils appeared to be disrupted by the surfactant, which eventually formed islands. Instead, cylindrical aggregates with an average size of 20–50 nm and a height of up to 8 nm were formed (Fig. 9C). It is similar that electrostatic repulsion between the individual (well exposed) micelles of C6IMIC12 contributed to the breakup of fibrillar forms and led to the formation of compact peptide aggregates.

#### Nuclear magnetic resonance ^1^H NMR

**Fig. 10:**
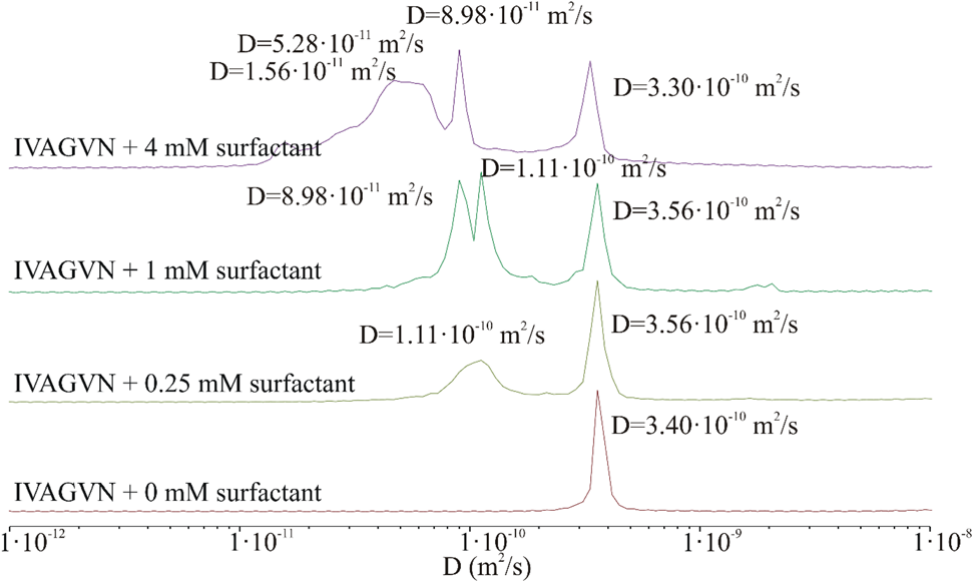
Protection of distribution of diffusion coefficient derived from 2D DOSY spectra of the peptide (0.52 mM) in the presence of different surfactant concentrations: 0 mM (red), 0.25 mM (light green), 1.00 mM (green) and 4.00 mM (purple).

The effect of the C6IMIC12 surfactant on the fibril formation was also monitored using diffusion-ordered spectroscopy (DOSY) (Fig. 10 and S3 in the ESI Material†). The addition of surfactants induced a polydisperse system with multicomponent diffusion. However, regardless of the surfactant concentration, the signal corresponding to the peptide diffusion coefficient (3.30 × 10^−10^ to 3.51 × 10^−10^ m^2^ s^−1^) appears in each mixture. This range of self-diffusion coefficient values suggests the presence of the peptide in a non-aggregated form. In turn, the remaining signals refer to particles with slower diffusion and consequently larger sizes. The values of these self-diffusion coefficients are typical for micelles rather than fibrils and are in good agreement with the values determined for C6IMIC12 itself.^49^ Only an increase in the surfactant concentration to 4.00 mM seems to induce the formation of elongated structures with a self-diffusion coefficient of ∼1.56 × 10^−11^ m^2^ s^−1^, which may be the seeds of fibrils. Calculating the hydrodynamic radius from NMR studies for a mixture of peptides and different surfactant concentrations was practically impossible. With such a dispersion of the diffusion coefficient and its wide band observed in Fig. 10, one can only state that the tested sample is inhomogeneous; it has aggregates of different sizes. This irregularity could result from interactions between the peptide and the surfactant, as well as interactions among the surfactant molecules themselves.

#### Circular dichroism

**Fig. 11:**
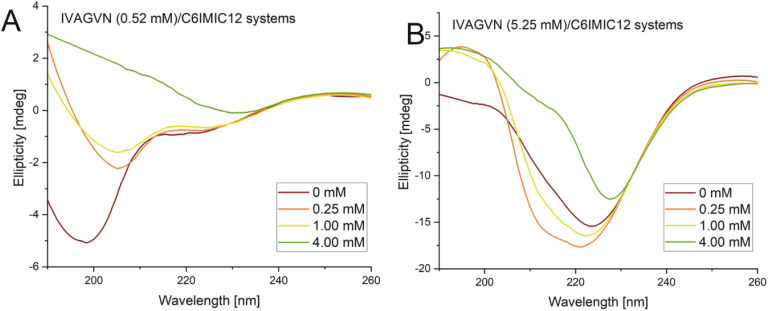
Circular dichroism spectra obtained for mixed systems IVAGVN/C6IMIC12, where peptide concentration was (A) 0.52 mM and (B) 5.25 mM with surfactant concentration range of 0–4.00 mM.

CD spectra collected for the IVAGVN peptide at 0.52 mM in the presence of increasing amounts of C6IMIC12 are presented in Fig. 11A. It is clearly visible that as the surfactant concentration increases, both minima become shallower and shift towards longer wavelengths. At higher surfactant concentrations (above 1.00 mM), the minimum at 203 nm disappears, and the spectra adopt the shape that suggests that the peptide primarily assumes an antiparallel β-sheet conformation.^41,42,50,51^ Calculations of the content of secondary structure elements indicated that the contribution of the β-sheet structure increased gradually from approximately 23% in the initial system to 47% at 4.00 mM (Table S2, ESI Material†). Concurrently, the contribution of the random coil decreased from 52% to *ca.* 30% at the highest surfactant concentration. When the peptide concentration was increased tenfold, the rising surfactant levels induced a distinct pattern of conformational changes. The structure of the peptide at a concentration of 5.25 mM transitioned from a random coil through a helical intermediate state (as evidenced by the broadening of the spectra towards shorter wavelengths and the appearance of a new, weakly defined band above 210 nm) and finally to an antiparallel left-hand twisted β-sheet (Fig. 11B).

According to the secondary structure content calculations, under initial conditions, the peptide was mostly unstructured (48%) with some contribution from β-sheet (parallel and antiparallel; 24%) and helical (9%) structures (Table S2, ESI Material†). The contribution of β-sheet structure(s) also increased to 32% at 1.00 mM surfactant concentration, reaching the maximum value of 31% at 4.00 mM.

#### Fourier transform infrared spectroscopy

Independently of CD analyses, the conformational changes in the studied mixed peptide/surfactant systems were also analysed using FTIR spectroscopy. However, to obtain IR spectra of satisfactory quality, it was necessary to carry out analyses in transmission mode for dried samples deposited on CaF_2_. This approach permits only qualitative analysis of changes, as the obtained samples are analysed in the dehydrated form. Nevertheless, the obtained results show a similar trend in secondary structure changes to those observed in circular dichroism spectroscopy studies.

**Fig. 12:**
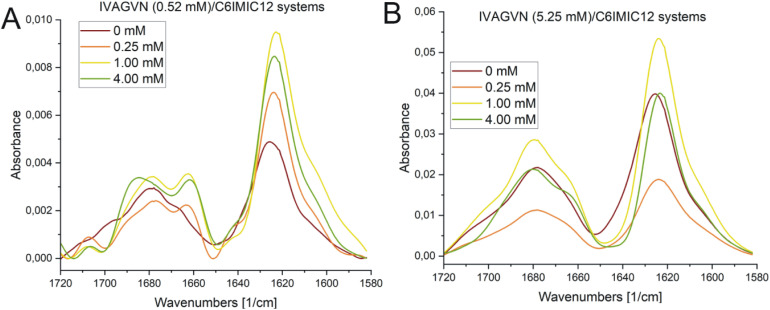
FTIR spectra obtained for IVAGVN/C6IMIC12 mixed systems, where the peptide concentration was (A) 0.52 mM and (B) 5.25 mM with surfactant concentration range of 0–4.00 mM.

The absorption peaks for the IVAGVN peptide at 0.52 mM were clearly visible on the FTIR spectrum, as presented in Fig. 12A. For both the peptide alone and in the presence of the surfactant, three dominant maxima were observed in the spectrum. The first and most intense peak at 1626 cm^−1^ is followed by bands at 1666 cm^−1^ and 1677 cm^−1^.^52^ All these bands suggest the presence of β-structures, including β-sheets: both antiparallel and parallel, intermolecular (aggregated) β-sheets and β-turns. The band *ca.* 1626 cm^−1^, exhibiting the highest intensity, is particularly noteworthy as it most likely originates from intermolecular β-sheets. Additionally, the antiparallel β-sheet is characterised by two bands: one with a weaker minimum of around 1670 cm^−1^ (observed in the spectra), and the second with a strong intensity of approximately 1630 cm^−1^. This latter band is likely shifted and overlaps with the signals from the intermolecular β-sheets and the parallel β-sheets (1638–1632 cm^−1^). The signal at 1666 cm^−1^ is also significant, as it likely corresponds to β-turns, which typically appear within the range of 1685–1655 cm^−1^. Of particular interest is the change in intensity in the 1630–1620 cm^−1^ region. For the pure peptide, this band shows a lower intensity and is closer to 1630 cm^−1^. However, with increasing surfactant concentration, there is a noticeable increase in the intensity of this band and a shift towards higher values, peaking around 1620 cm^−1^ at a concentration of 1.00 mM. This suggests that the addition of surfactant promotes the formation of intermolecular β-sheets, up to a concentration of 1.00 mM. Furthermore, the surfactant appears to induce a more pronounced maximum at around 1660 cm^−1^, which can be attributed to the enhanced formation of β-turns. FTIR absorption spectra recorded for the IVAGVN/C6IMIC12 systems for peptide at 5.25 mM concentration and peptide/surfactant molar ratios from 20 : 1 to 1.25 : 1 show three well-developed maxima corresponding to the vibrations of the amide bond (Fig. 12B). For all investigated fibrils, the IR spectra are dominated by fibrillar β-sheet peaks around 1626 cm^−1^ and 1678 cm^−1^. The addition of surfactant induces changes in the spectrum across various regions. The intensity of the band at ∼1626 cm^−1^ decreases at low surfactant concentrations and then increases, and ultimately at concentrations above 1.00 mM, the spectrum resembles that of the pure peptide. Notably, a positive maximum at 1667 cm^−1^ appears only after the addition of the surfactant irrespective of its concentration. This band is generally assigned to β-turns.^52–54^

## Discussion

Proteins and peptides, as essential organic macromolecules, form the material basis of life and serve as the primary drivers of biological processes. Research into the mechanisms of aggregation and fibrillization of these molecules is a crucial component of understanding life at the molecular level, particularly in the context of neurodegenerative diseases. Despite decades of investigation, these processes remain insufficiently understood, and no comprehensive mechanism has yet been established to fully explain their occurrence. It has been proposed that surfactants, owing to their unique amphiphilic structure, can enhance the solubility of biological compounds, thereby reducing aggregate formation: a factor that could play a crucial role in the design and development of new drugs. Moreover, as demonstrated by other studies, surfactants may facilitate the disassembly of existing aggregates. Surfactants have already found extensive applications in the study of membrane protein structures^55^ and in areas such as drug delivery,^56^ disease treatment,^57^ and other medical fields, including research related to SarsCov2.^58^ A systematic investigation into surfactant-protein/peptide interactions holds the potential to offer valuable insights and significantly advance the broader field of life sciences.

The IVAGVN peptide, derived from an amyloidogenic protein, cystatin C is composed of six amino acid residues of which only one, an asparagine, exhibits hydrophilic character. The remaining amino acids are hydrophobic. The studies conducted by Iłowska *et al.* indicated that short fragments of cystatin can initiate the fibrilization and aggregation process.^22^ They demonstrated that peptides capable of forming a steric zipper motif display a high propensity to form fibrils or aggregates. The studied compound was shown to form oligomers contingent upon both its concentration and buffer conditions (type and pH). In the presented studies, the effect of peptide concentration was examined in greater detail. Within concentrations ranging from 0.52 mM to 2.60 mM, an increase in the concentration resulted in the formation of larger and more complex aggregates. Notably, a concentration of 2.60 mM deserves special attention, as small fibrils began to form under these conditions. This concentration also represents the threshold at which conformational changes, detectable *via* circular dichroism spectroscopy, start to emerge.

To better understand the influence of surfactants on the self-association of peptides, the effect of the gemini surfactant 3,3′-[1,8-(2,7-dioxahexane)]bis(1-dodecylimidazolium) chloride (C6IMIC12) on the aggregation process of IVAGVN was studied. The microscopic morphology of the samples (TEM and AFM) was examined for peptides at low concentrations (0.52 mM, Fig. 8 and 9), making it difficult to discuss the direct influence of the surfactant on the peptide aggregation process as a function of its concentration. These studies revealed that the addition of a small amount of surfactant (up to twice the peptide concentration) promotes aggregation. However, it remains unclear whether the observed aggregates originate solely from the peptide or include surfactant aggregates. This ambiguity highlights the complexity of peptide–surfactant interactions. The surfactant used in the studies can form micelles, as confirmed by Szutkowski *et al.*, who reported critical concentration (CC) values without explicitly designing them as CAC or CMC.^49^ For surfactant concentrations below the critical micelle concentration (approximately 0.50 mM), where surfactant micelles are absent, individual surfactant molecules can interact with peptides through hydrophobic interactions. These interactions arise from the attraction between the hydrophobic tails (hydrocarbon chains) of the gemini surfactant and the hydrophobic amino acid residues of the peptide. This type of interaction is expected to promote a peptide structure that is more exposed to the aqueous environment than its conformation in a surfactant-free solution. Additionally, the charged head groups of the gemini surfactant should effectively prevent peptide aggregation in such systems. In the absence of the surfactant, the hydrophobic regions of the peptide are likely to “coil” within the molecule, avoiding contact with water and forming steric zipper motifs. Thus, below the CMC of the gemini surfactant (C6IMIC12), the solubility of the IVAGVN peptide is theoretically expected to increase, while its tendency to aggregate should decrease relative to its behaviour in the absence of C6IMIC12.

A higher surfactant concentration (4.00 mM) led to a significant reduction of peptide aggregates; an effect that is distinctly visible in the microscopic images. These observations highlight the structural properties of the surfactant and its hydrophobic and electrostatic interactions with specific peptide residues, as well as the role of surfactant concentration in modulating aggregation. This sheds light on the intricate balance between promoting and inhibiting aggregate formation. At low molar ratios of the peptide to gemini surfactant, the surfactant stabilizes the peptide's secondary structure, as evidenced by CD and FTIR spectra (Fig. 11A and 12A), whereas higher molar ratios destabilised the structure. At surfactant concentrations exceeding the CMC, surfactant micelles are present in the system, significantly influencing the distribution of peptides in the solution. The IVAGVN peptide may adsorb onto the surface of these micelles, with its hydrophobic fragments embedding into their hydrophobic core and hydrophilic regions exposed to the aqueous environment. The local aggregation of the peptide on the surface of the micelle can promote interactions between peptide molecules, potentially leading to ordered aggregation, such as the formation of β-sheet structures. Near the CMC, the formation of micelles can potentially promote the local aggregation of peptides, favouring the development of ordered structures. At concentrations above the CMC, peptide aggregates may be disrupted, or the aggregation of IVAGVN peptides could be significantly reduced. Peptide molecules dissolved in surfactant micelles or aggregates have a reduced chance of self-association. Thus, high surfactant concentrations may prevent aggregation by stabilizing individual peptides within micelles. Similar interactions have been reported for other proteins. Li *et al.* and Wang *et al.*, who studied interactions between haemoglobin, BSA and gemini surfactants.^59–61^ Their research confirmed the presence of spherical complexes and demonstrated that surfactants facilitate the formation of larger aggregates up to a certain concentration. Beyond this point, the aggregates diminished and were reduced. Comparing the observed behaviour of the studied IVAGVN peptide–surfactant systems with other model systems based on amyloidogenic peptides, it is noteworthy that β-sheet rich peptides are often more resistant to the denaturation process than α-helical peptides.^62,63^ The most frequently conducted studies of peptide–surfactant interactions focused on systems based on the most popular model amyloidogenic peptide-Aβ. Various surfactants have been tested for systems based on different forms of Aβ, including both monomeric anionic^64^ and cationic surfactants, zwitterionic surfactants, oligomeric surfactants^65^ and phospholipids.^66^ For the Aβ(1–40) in the presence of a small amount of dicationic gemini surfactant C_6_C_12_C_6_Br_2_, a significant increase in the β-sheet structure was observed.^37^

At higher peptide concentrations (5.25 mM), no significant structural changes were observed irrespective of the surfactant concentration, as confirmed by the CD and FTIR spectra (Fig. 11B and 12B). Microscopic imaging was also rendered impossible due to the excessive aggregation of both the peptide and surfactant. This suggests that the predominant peptide concentration restricts the surfactant's capability to modify the aggregation dynamics, with the excessive aggregation of both the peptide and surfactant hindering further microscopic analysis. Future studies will aim to explore this process using peptide-to-surfactant molar ratios similar to those used for the 0.52 mM peptide concentration. However, such an approach might involve risks because very high surfactant concentrations (up to 42 mM) could lead to the study of two independent auto-aggregation processes: one for the IVAGVN peptide and the other for the surfactant, rather than their interactions.

Srivastava and Alam investigated the interactions between gemini surfactant (1,6-bis(*N*,*N*-hexadecyl dimethyl ammonium) bromide: 16-6-16) and human serum albumin (HSA).^67^ They demonstrated that these interactions are significantly influenced by the size and charge of surfactant aggregates and depend on both the concentrations of the individual molecules and the pH of the experimental buffer. Similar to our study, they employed AFM, CD, and molecular docking techniques to test their hypotheses. They observed notable conformational changes in HSA upon interaction with 16-6-16. At pH 4.0 (*i.e.* below the protein's isoelectric point, PI 4.7), native HSA exhibited a predominantly helical conformation (up to 50%). Upon the addition of surfactant, the helicity decreased, and β-sheet content increased. A similar effect was observed at a pH above the PI value. At a pH equal to the PI (4.7), complete CD spectra measurements were hindered by turbidity, preventing the analysis of all solutions. Nevertheless, AFM analysis confirmed that the level and nature of aggregation vary with surfactant concentration, which is similar to our study. Consequently, dimeric surfactants, such as 16-6-16, were shown to induce the unfolding and aggregation of the protein. These data, even though obtained for a full-length protein, can be related to our case, where peptide aggregational behaviour is also modulated by gemini surfactant.

## Experimental methods

### Materials

**Fig. 13:**
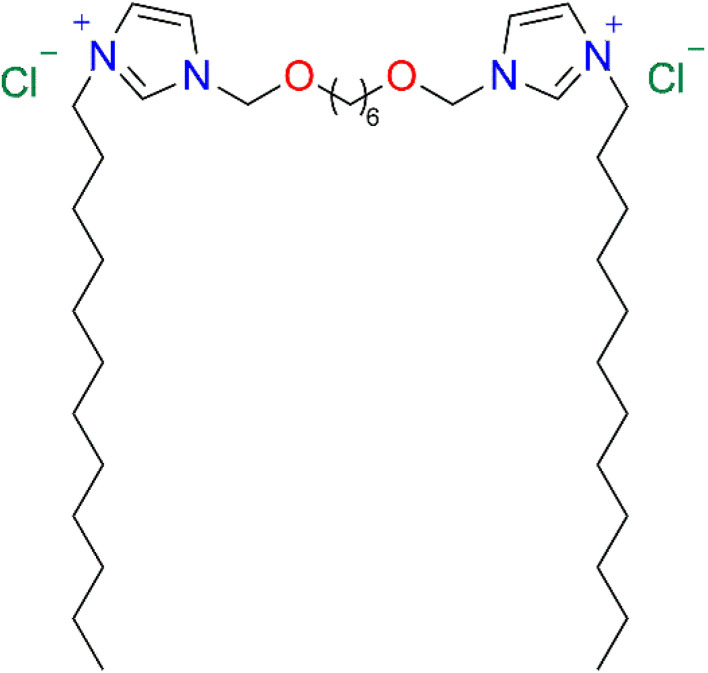
Gemini surfactant-3,3′-[1,8-(2,7-dioxahexane)]bis(1-dodecylimidazolium) chloride (C6IMIC12).

The IVAGVN peptide was synthesised using the previously described methodology.^22^ Gemini surfactant-3,3′-[1,8-(2,7-dioxahexane)]bis(1-dodecylimidazolium) chloride (C6IMIC12) (Fig. 13) was a generous gift from Professor Andrzej Skrzypczak‡‡Andrzej Skrzypczak passed away September 18th 2023. (Faculty of Chemical Technology, Poznań Technical University, Poland). The CAC value of this surfactant was 0.47 mM.^49^ Milli-Q water was used throughout the study. For NMR DOSY experiments, a deuterium oxide was used.

### Sample preparation

In experiments examining the effect of peptide concentration on its aggregation, the peptide was dissolved in 10 mM PBS at pH 7.4 to final concentrations of 0.52 mM, 1.75 mM, 2.60 mM, 3.50 mM and 5.25 mM. In the experiments assessing the impact of the gemini surfactant on the peptide aggregation characteristics, the peptide solutions in PBS at pH 7.4 were mixed with the surfactant solutions to give the final peptide concentration of 0.52 mM (low peptide concentration) or 5.25 mM (high peptide concentration) and the surfactant concentrations of 0.25 mM; 1.00 mM and 4.00 mM, respectively. These samples were analysed using the techniques described below.

### Computer modelling and docking

The modelling and docking of the peptide to the surfactant molecule were performed using the YASARA 24.10.5 software^68^ and AutoDock Vina.^69^ Molecular dynamics (MD) simulations were conducted for a duration of 80 nanoseconds (ns) using a system comprising around 50 000 atoms, including 45 peptide molecules and water molecules (TIP3P model). The physiological conditions were maintained at a sodium chloride concentration of 0.9% by weight, and the pH was adjusted to 7.7. The system was maintained at a temperature of 298 kelvin, and constant pressure and temperature were maintained using the NPT ensemble.^70^ The periodic boundary conditions (PBCs) were implemented with a cut off distance of 12 angstroms, and the particle-mesh Ewald method was employed for Coulomb interactions. The AMBER15IPQ force field was utilized. The analysis of the secondary structure was performed using the built-in YASARA macro (MD_analyze). The mean-square-displacement (MSD) analysis in six directions (*M*_*xx*_, *M*_*yy*_, *M*_*zz*_, *M*_*xy*_, *M*_*xz*_, *M*_*yz*_) was derived from the trajectory for carbon atoms using the Python MDAnalysis library. The diffusion tensor components (*D*_*xx*_, *D*_*yy*_, *D*_*zz*_, *D*_*xy*_, *D*_*xz*_, *D*_*yz*_) were subsequently calculated from the slopes using the Stokes–Einstein relation (MSD = 2*Dt*).

### Transmission electron microscopy

10 μl of peptide solutions or peptide mixture with a surfactant, prepared as described above (Sample preparation), was placed on formvar/carbon supported copper grids (TEM Pelco 300 Mesh Grids, Ted Pella, Inc., USA) and allowed it to adsorb for 30 seconds. Then, the liquid was removed from the grid using filter paper strips. A drop of water was then placed on the grid and immediately removed using filter paper strips. Finally, 5 μl of 2% (v:v) uranyl formate was applied to the samples. After 30 seconds, it was removed from the grid with filter paper strips. TEM images were recorded using a JEM-1400 transmission electron microscope (Jeol JEM-1400, series 120 kV).

### Atomic force microscopy

AFM analyses of IVAGVN solutions, both alone and in the presence of gemini surfactant, were conducted using the JPK NanoWizard® 4 atomic force microscope (JPK Instruments, Germany). Samples prepared as described above were diluted 1 : 200 using MQ water. 10 μl of samples were deposited on a freshly cleaved mica surface and dried overnight before visualization. The measurements were performed in QI (Quantitative Imaging Mode) using Tap150AL AFM cantilevers (Ted Pella, Inc., USA). All topographic scans were analysed using the Gwyddion 2.49 software.^71^

### ^1^H NMR diffusometry

The self-diffusion data were obtained using a high resolution NMR 600 MHz spectrometer (Agilent) equipped with a DSI-1374 gradient probe head adapted for temperature-dependent measurements. A DOSY experiment with a pulsed field gradient in a stimulated spin echo sequence (PFGSTE) and with pre-saturation of the water line was used for the FID registration. The magnetic field was stabilised using a 2H lock throughout all experiments. The parameters for the DOSY sequence were set as follows: diffusion time, *Δ* = 50 ms; gradient duration, *δ* = 2 ms; gradient intensity *g*-varied in range from 1.77 Gs cm^−1^ to 250 Gs cm^−1^ in 32 steps. All samples were measured at 25 °C with a repetition time of 5 s. The number of scans, depending on the sample concentration, was set as a multiple block of 32 repetitions. The samples were prepared in standard 5 mm HR NMR sample tubes shortly before the start of the experiment. The collected raw data were transferred into the MestReNova for processing. After standard spectra preparation, the DOSY spectra were calculated using the Bayesian DOSY transform tool of the MestReNova package.^72^

### Circular dichroism

The CD spectra in the far UV region (190–260 nm) were recorded using a Jasco J-815 spectropolarimeter (Jasco, Canada) at 20 °C. Sample solutions were placed into a demountable quartz cuvette (Helma, Germany) with a 0.5 mm pathlength. Five accumulations were collected and averaged for each CD spectrum recorded at a scanning speed of 50 nm min^−1^. The obtained spectra were deconvoluted using the BeStSel programme with the reference set SP175.^73^

### Fourier transform spectroscopy

The secondary structure of the IVAGVN peptide at a concentration of 0.52 mM or 5.25 mM, both alone and in the presence of the C6IMIC12 surfactant at selected concentrations (see the Sample preparation section above), was also assessed using FTIR spectra recorded at room temperature using a Tensor 27 FTIR spectrometer (Bruker-Optics, Germany). The samples (10 μl) were placed on a CaF_2_ window and allowed to dry. Subsequently, the FTIR spectra were measured in transmittance mode in a wavenumber ranging from 4000 cm^−1^ to 1000 cm^−1^. For each sample, 256 interferograms were collected at a spectral resolution of 2 cm^−1^. The baseline correction (using adaptive settings: coarseness 15 and offset −5), smoothing (Savitzky–Golay interval 35, polynomial order 5), transformation for absorbance, and cutting (1580–1720 nm) for all spectra were done using Spectragryph v1.2.16.1 free software.^74^

## Conclusions

In this study, we demonstrated that the concentration of the IVAGVN peptide, designed based on the human cystatin C sequence and the presence of gemini surfactant, can significantly influence the secondary structure of the peptide and the morphology of its aggregates. We showed that the formation and characteristics of peptide aggregates are associated with the concentration of the peptide as well as the surfactant-to-peptide ratio. At lower peptide concentrations, small aggregates are formed, while at higher concentrations, they tend to interact with each other, leading to the formation of more cumulative forms. The addition of gemini surfactant C6IMIC12 modulates the aggregation characteristics of IVAGVN. As the surfactant concentration increases, the peptide forms aggregates composed of loosely connected micelles. Above the CMC of the surfactant, β-sheet content increases, suggesting the formation of more ordered fibrils.

## Data availability

All data obtained during the study and used in this manuscript (raw data) are available upon request from the leader of the project (Julia Ludwiczak, julia.maria.ludwiczak@gmail.com) or corresponding author (Emilia Iłowska, emilia.ilowska@ug.edu.pl). Data sets supporting the conclusions and descriptions of the complete protocols can be found in the manuscript and its ESI files.†

## Author contributions

Conceptualization, J. L., E. I., M. W. and M. Kozak; methodology, J. L., M. W.; validation, J. L., M. D., E. I.; formal analysis, E. I., A. S., J. L; investigation, J. L., M. Kempka, A. S., E. I., K. S.; data curation, J. L.; writing—original draft preparation, J. L., E. I., A. S., K. S.; writing—review and editing, all authors; visualization, J. L., E. I., K. S.; supervision, M. Kozak; project administration, J. L.; funding acquisition, J. L.

## Conflicts of interest

The authors declare no conflict of interest.

## Supplementary Material

RA-015-D4RA08377F-s001
