# Supplementary material for: The influence of a dicationic surfactant on the aggregation process of the IVAGVN peptide derived from the human cystatin C sequence (56–61)
Source: RSC Adv. 2025 Jan 31;15(5):3237–49. doi: 10.1039/d4ra08377f (PMC11784886; doi:10.1039/d4ra08377f)
Supplement: RA-015-D4RA08377F-s001 [file RA-015-D4RA08377F-s001.pdf]

Supplementary Information (SI) for RSC Advances.
This journal is © The Royal Society of Chemistry 2025

The influence of dicationic surfactant on the aggregation process of the IVAGVN peptide from human cystatin C sequence (56-61)

Julia Ludwiczak[a], Emilia Iłowska[b*], Michalina Wilkowska[a], Aneta Szymańska[c], Marek Kempka[a], Maria Dobies[a], Kosma Szutkowski[d], Maciej Kozak[a]

[a.] *Department of Biomedical Physics, Adam Mickiewicz University, Poznan, Poland.*
[b.] *Department of Organic Chemistry, University of Gdansk, Gdansk, Poland. emilia.ilowska@ug.edu.pl*
[c.] *Department of Biomedical Chemistry, University of Gdansk, Gdansk, Poland.*
[d.] *NanoBiomedical Centre,, Adam Mickiewicz University, Poznan, Poland.*
** Corresponding author: emilia.ilowska@ug.edu.pl*

Table S1. Contribution [%] of secondary structure elements determined from CD spectra for different peptide concentrations [mM]. 2

Table S2. Contribution [%] of secondary structure elements determined from CD spectra for different peptide concentrations: 0.52 and 5.25 mM and with C6IMIC12 surfactant with various concentrations (0.25, 1.00 and 4.00 mM). 3

Figure S1. A) The mean-square displacement (MSD) analysis for the 0-80 ns interval and B) the decomposition of MSD into its matrix components (MSD_{xx}, MSD_{yy}, MSD_{zz}, MSD_{xy}, MSD_{xz}, MSD_{yz}), diffusion tensor, diffusion tensor eigenvalues (l_1, l_2, l_3) and resulting mean diffusion coefficients. 4

Figure S2. DOSY ^{1}H NMR spectra for diffusion coefficient measurements of the peptide IVAGVN at various concentrations: 0.52 mM; 1.05 mM, 1.75 mM. 3.50 mM and 5.25 mM. 7

Figure S3. DOSY ^{1}H NMR spectra for diffusion coefficient measurements of the peptide IVAGVN (0.52 mM) with the presence of different surfactant concentrations: 0 mM, 0.25 mM, 1.00 mM, 2.00 mM and 4.00 mM. 10

Table S1. Contribution [%] of secondary structure elements determined from CD spectra for different peptide concentrations [mM].

C_{IVAGVN} [mM]	a-helix [%]	b-sheets [%]	Turns [%]	Unordered [%]
0.52	7.8	23.5	16.6	52.1
1.05	9.3	17.9	17.8	55
2.60	16.4	22.8	16.4	44.5
3.50	14.9	23.2	16	45.9
5.25	9.4	24.3	18.3	48.1

Table S2. Contribution [%] of secondary structure elements determined from CD spectra for different peptide concentrations: 0.52 and 5.25 mM and with C6IMIC12 surfactant with various concentrations (0.25, 1.00 and 4.00 mM).

C_{IVAGVN} [mM]	$C_{surfactanct}$ [mM]	a-helix [%]	β-sheets [%]	Turns [%]	Unordered [%]
0.52	0	7.8	23.5	16.6	52.1
	0.25	4.5	29.3	19.0	47.2
	1.00	7.9	27.4	16.1	48.9
	4.00	0	47.2	23.2	29.3
5.25	0	9.4	24.3	18.3	48.1
	0.25	15.2	25.8	14.6	44.4
	1.00	7.5	32.8	15.3	44.4
	4.00	7.1	31.0	20.7	41.2

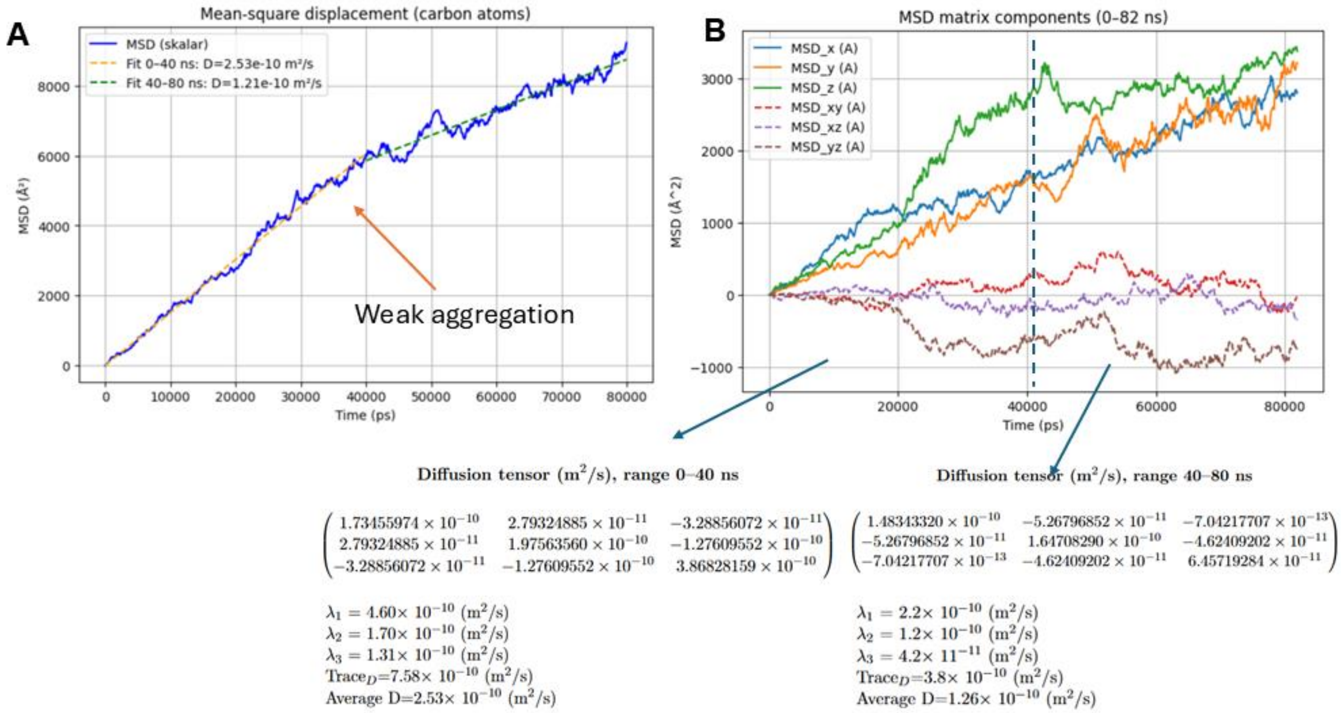

Figure S1. A) The mean-square displacement (MSD) analysis for the 0-80 ns interval and B) the decomposition of MSD into its matrix components (MSD_{xx}, MSD_{yy}, MSD_{zz}, MSD_{xy}, MSD_{xz}, MSD_{yz}), diffusion tensor, diffusion tensor eigenvalues (l_1, l_2, l_3) and resulting mean diffusion coefficients.

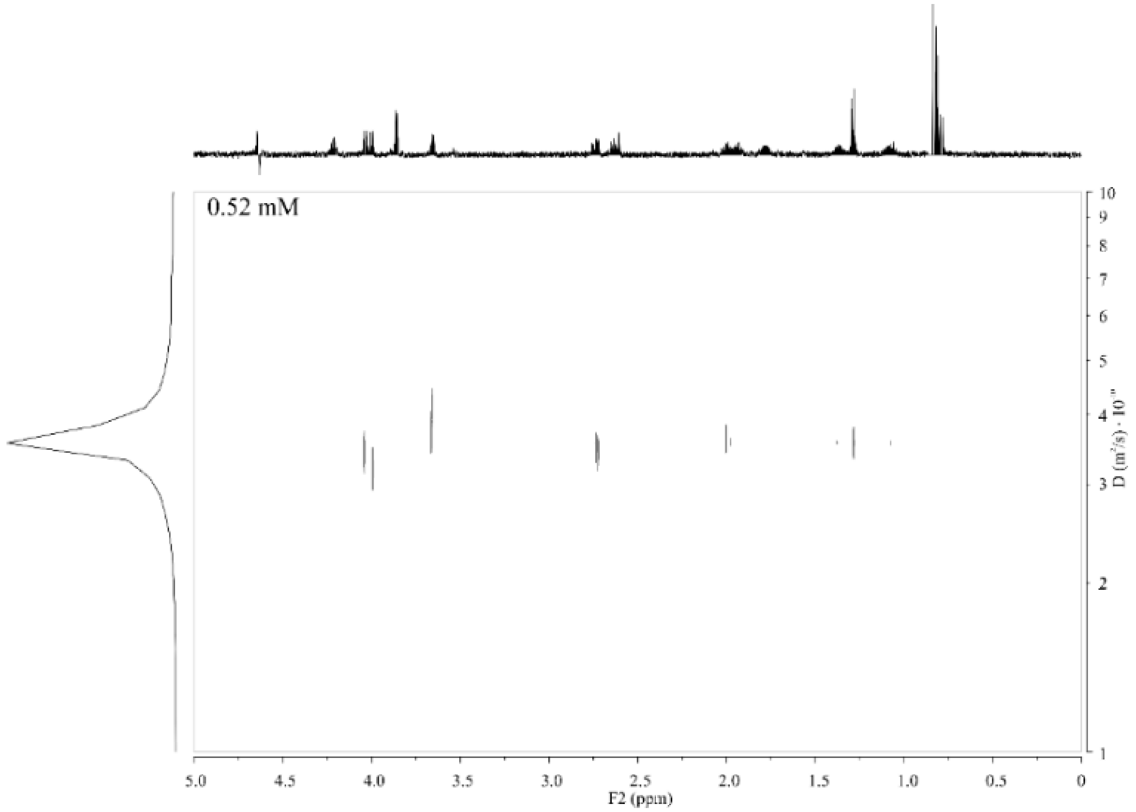
0.52 mM
F2 (ppm)

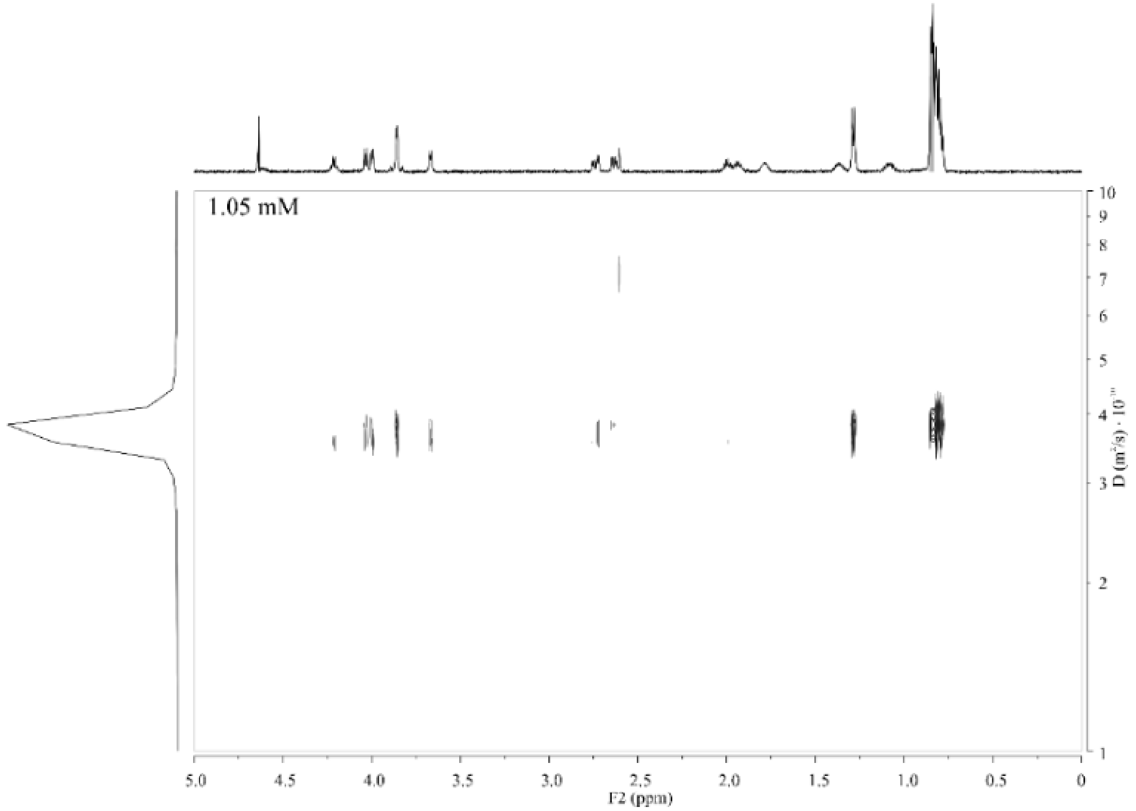
1.05 mM
F2 (ppm)

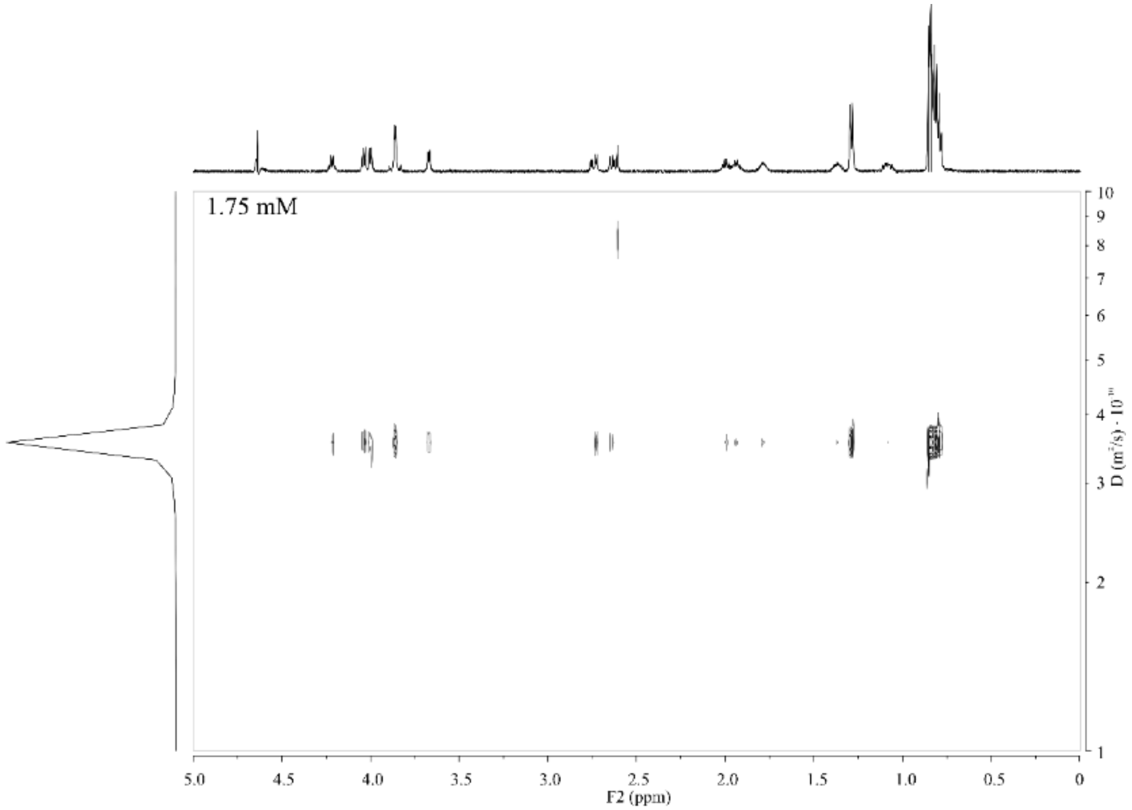

1.75 mM
D (m²/s) · 10⁻¹⁰
F2 (ppm)

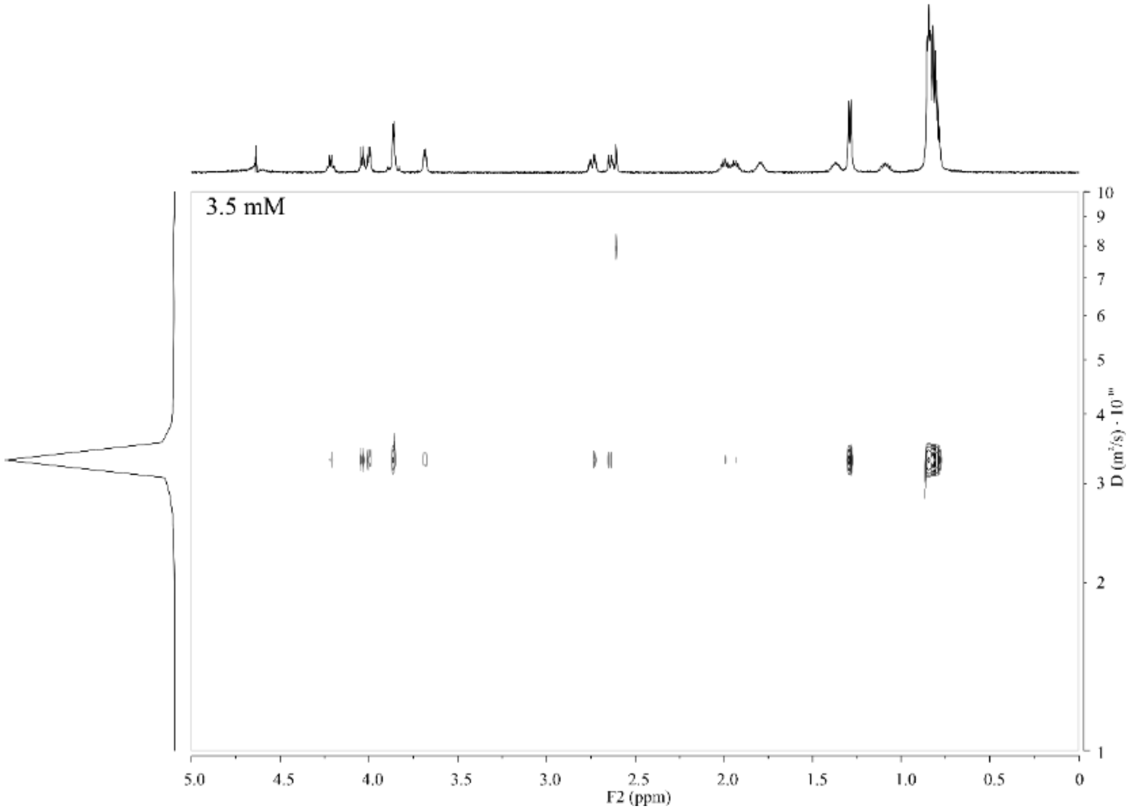

3.5 mM
D (m²/s) · 10⁻¹⁰
F2 (ppm)

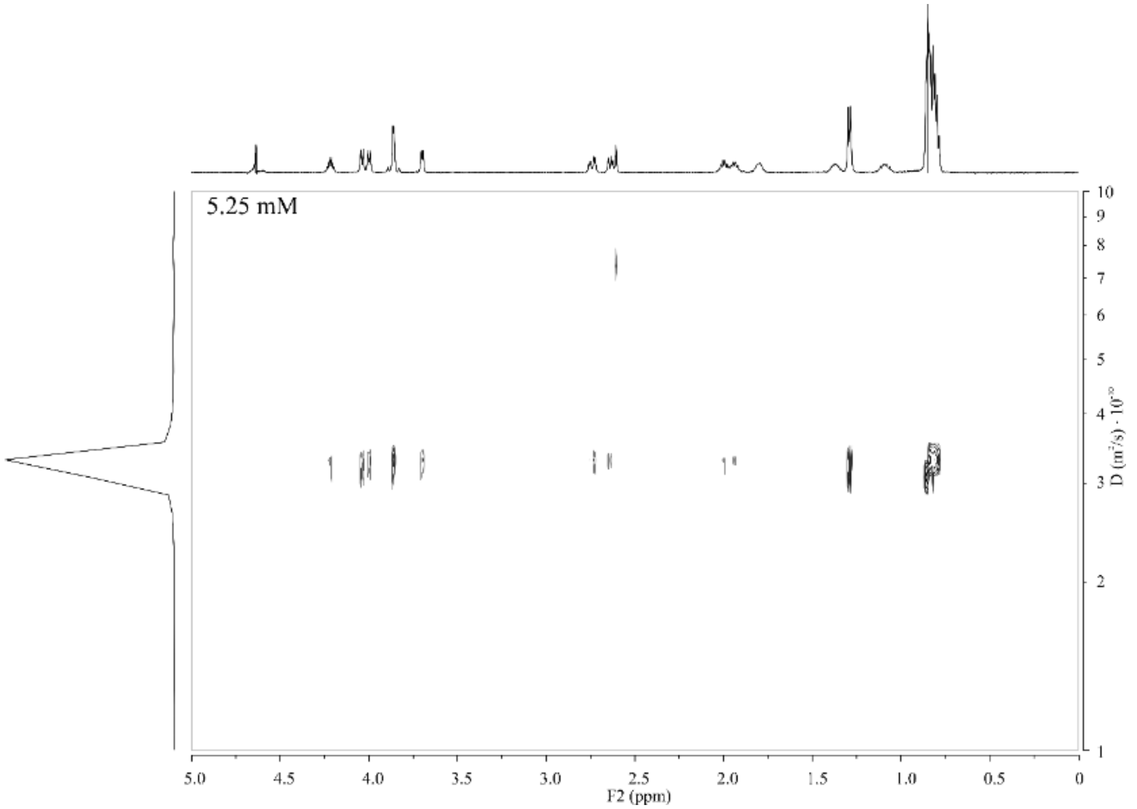

Figure S2. DOSY ^{1}H NMR spectra for diffusion coefficient measurements of the peptide IVAGVN at various concentrations: 0.52 mM; 1.05 mM, 1.75 mM. 3.50 mM and 5.25 mM.

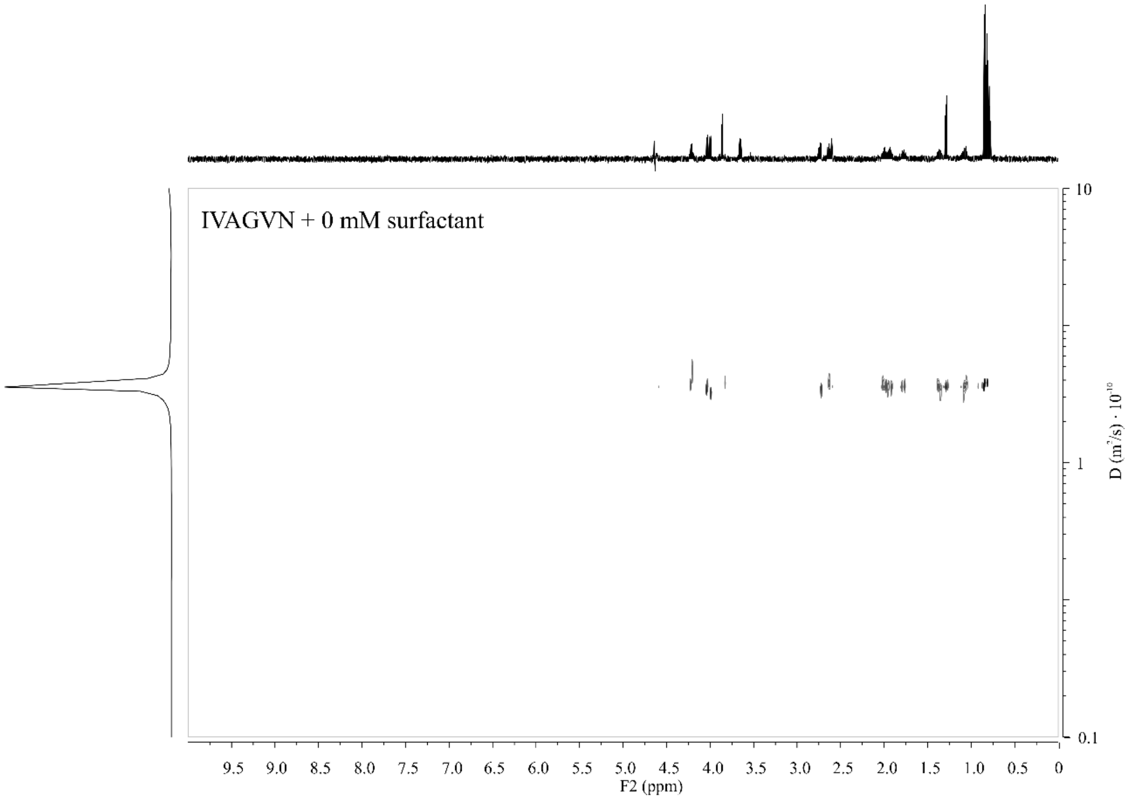
IVAGVN + 0 mM surfactant
10
1
0.1
D (m²/s) · 10⁻¹⁰
9.5 9.0 8.5 8.0 7.5 7.0 6.5 6.0 5.5 5.0 4.5 4.0 3.5 3.0 2.5 2.0 1.5 1.0 0.5 0
F2 (ppm)

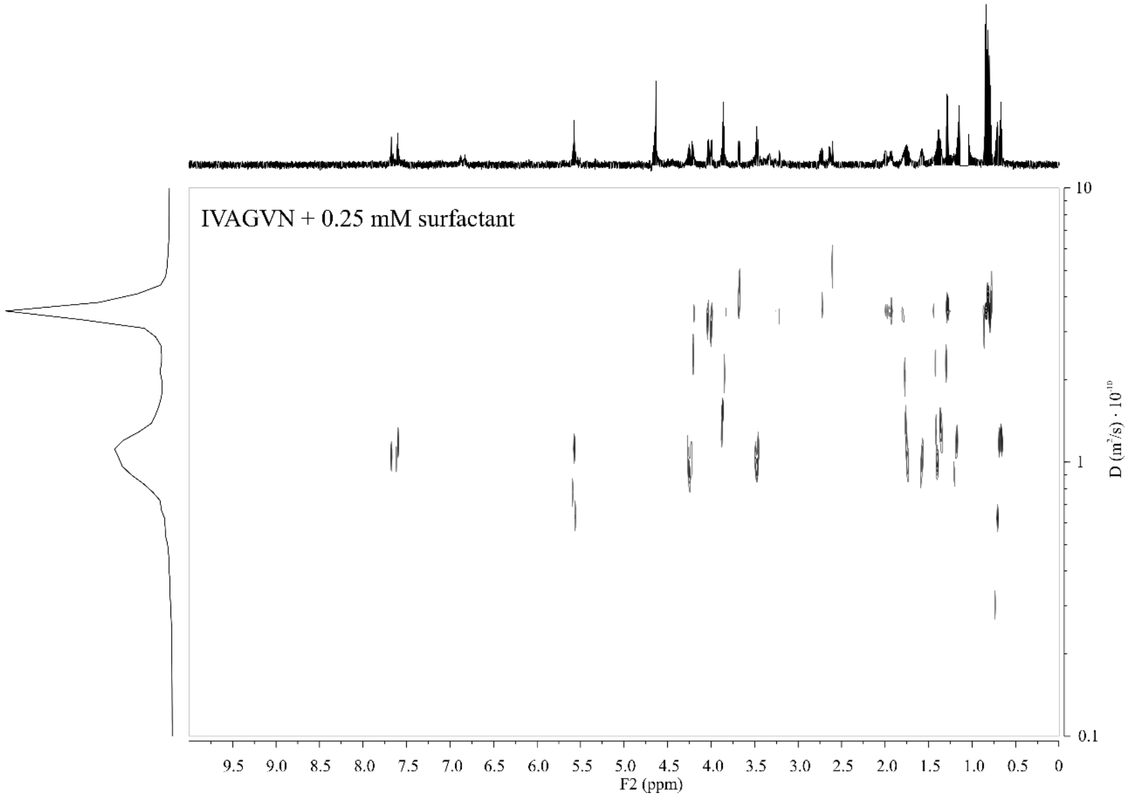
IVAGVN + 0.25 mM surfactant
10
1
0.1
D (m²/s) · 10⁻¹⁰
9.5 9.0 8.5 8.0 7.5 7.0 6.5 6.0 5.5 5.0 4.5 4.0 3.5 3.0 2.5 2.0 1.5 1.0 0.5 0
F2 (ppm)

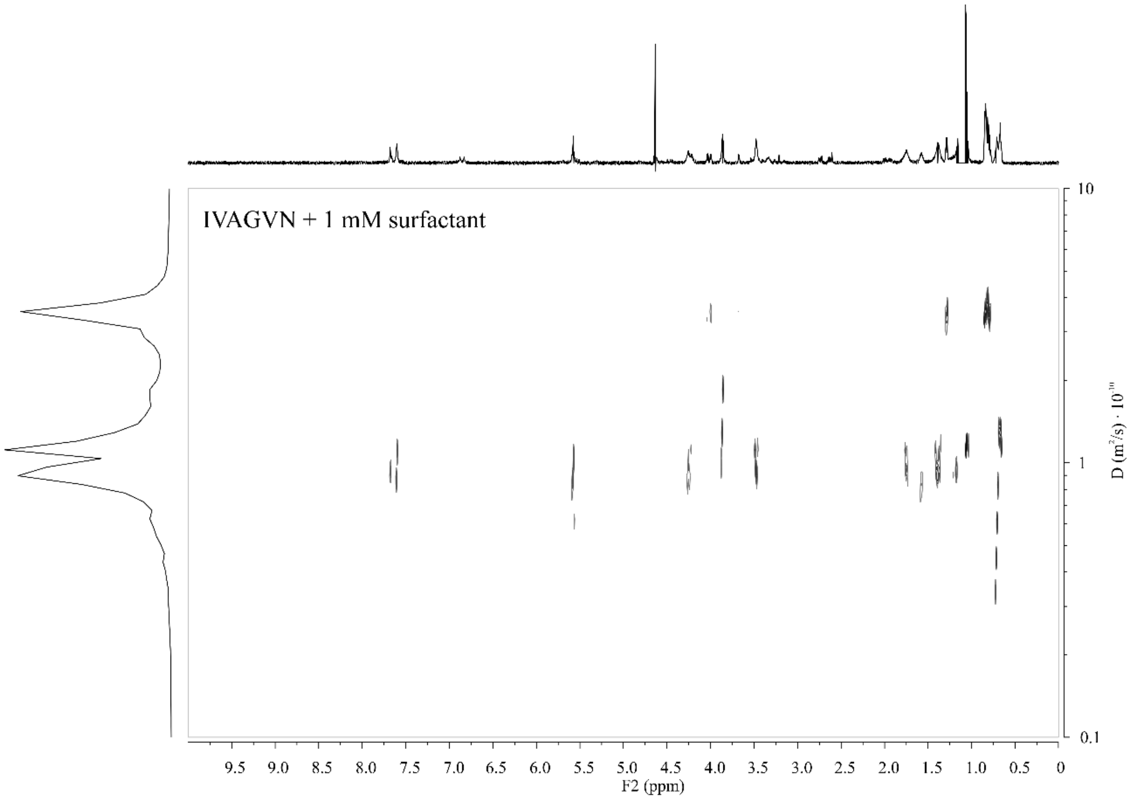
IVAGVN + 1 mM surfactant
D (m²/s) · 10⁻¹⁰
10
1
0.1
9.5 9.0 8.5 8.0 7.5 7.0 6.5 6.0 5.5 5.0 4.5 4.0 3.5 3.0 2.5 2.0 1.5 1.0 0.5 0
F2 (ppm)

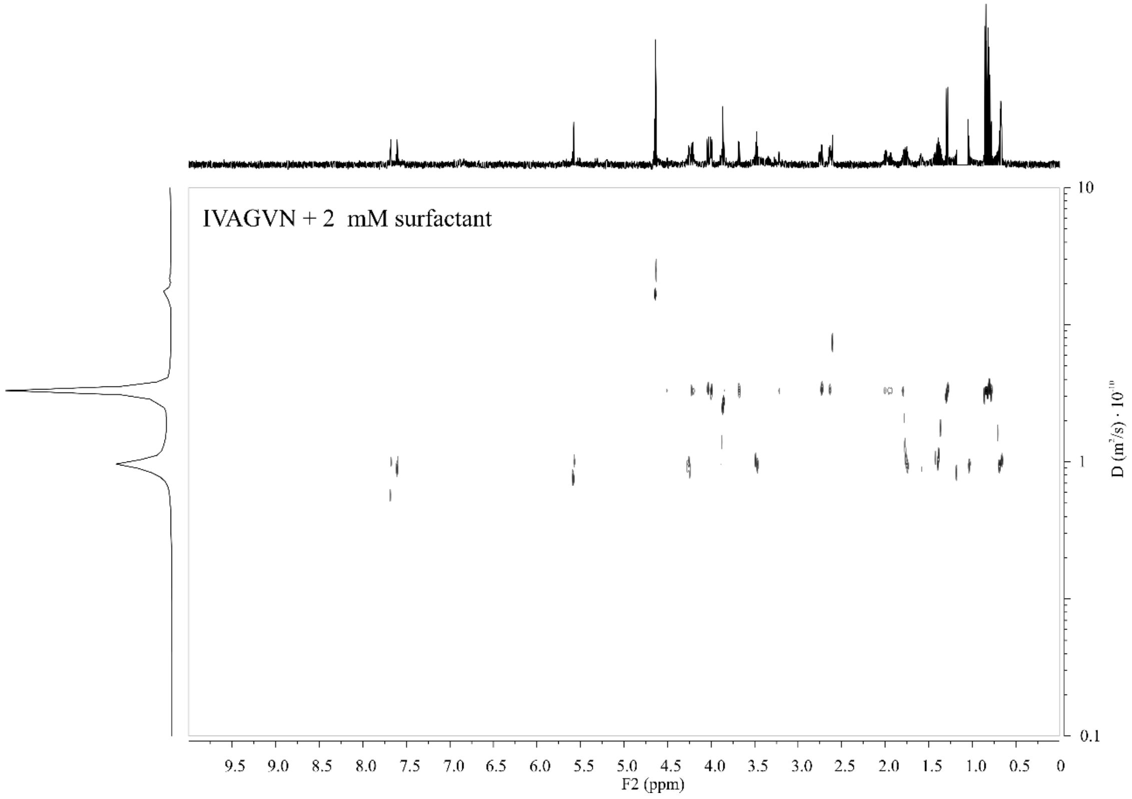
IVAGVN + 2 mM surfactant
D (m²/s) · 10⁻¹⁰
10
1
0.1
9.5 9.0 8.5 8.0 7.5 7.0 6.5 6.0 5.5 5.0 4.5 4.0 3.5 3.0 2.5 2.0 1.5 1.0 0.5 0
F2 (ppm)

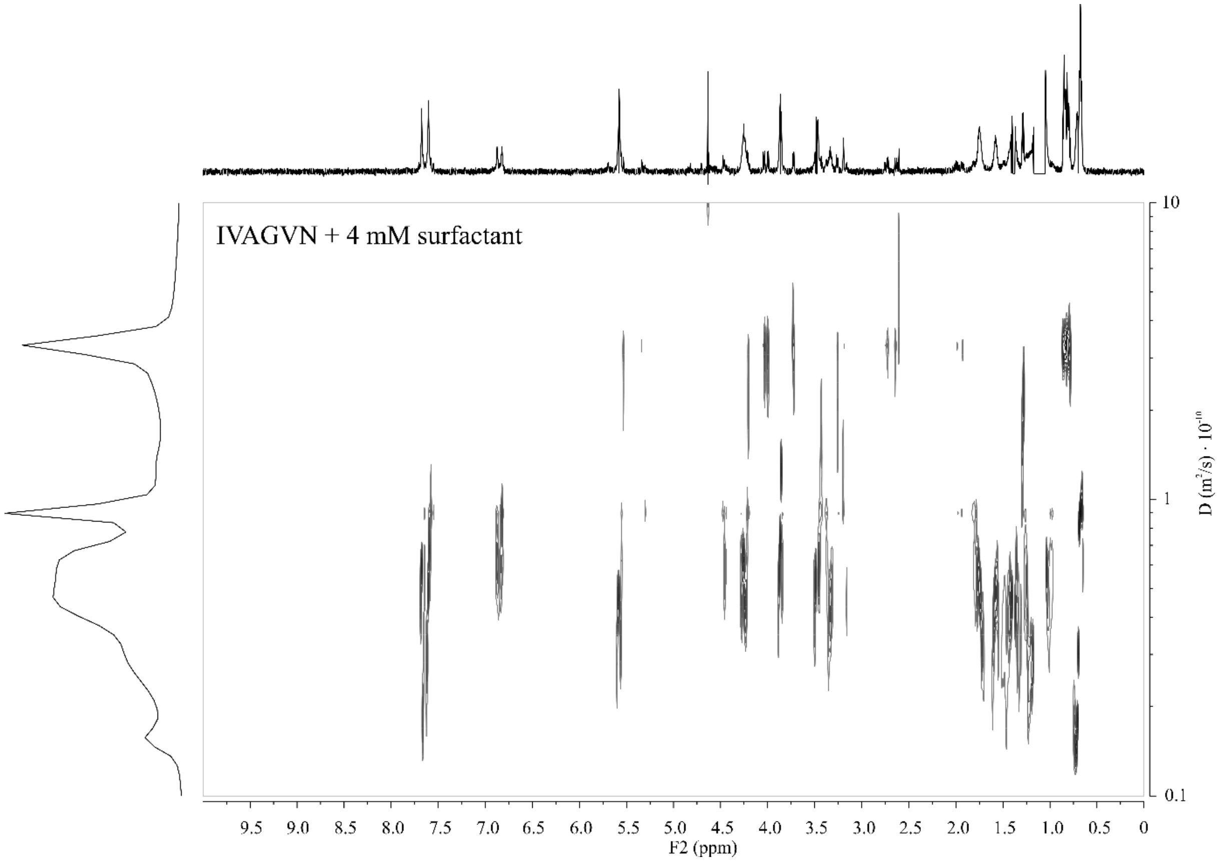

Figure S3. DOSY ^{1}H NMR spectra for diffusion coefficient measurements of the peptide IVAGVN (0.52 mM) with the presence of different surfactant concentrations: 0 mM, 0.25 mM, 1.00 mM, 2.00 mM and 4.00 mM.
